# Biochemical, physiological, and performance response of a functional watermelon juice enriched in L-citrulline during a half-marathon race

**DOI:** 10.1080/16546628.2017.1330098

**Published:** 2017-06-13

**Authors:** Ascensión Martínez-Sánchez, Domingo J. Ramos-Campo, Bárbara Fernández-Lobato, Jacobo A. Rubio-Arias, Fernando Alacid, Encarna Aguayo

**Affiliations:** ^a^ Food Quality and Health Group, Institute of Plant Biotechnology, Universidad Politécnica de Cartagena, Campus Muralla del Mar, Cartagena, Spain; ^b^ Department of Physical Activity and Sport Science, Faculty of Sport, Catholic University of Murcia (UCAM), Murcia, Spain; ^c^ Pharmacy Department, Hospital General Universitario Santa Lucía, Cartagena, Spain

**Keywords:** Ergogenic supplements, lactate dehydrogenase, lactate, muscle soreness, glucose, arginine

## Abstract

**Background**: Watermelon is a rich natural source of l-citrulline. This non-essential amino acid increases exercise performance.

**Objective**: Evaluate the effect of Fashion watermelon juice enriched in l-citrulline (CWJ) (3.45 g per 500 mL) in physical performance and biochemical markers after a half-marathon race.

**Design**: A randomised, double blind, crossover design where 2 h after drinking 500 mL of CWJ or placebo (PLA, beverage without l-citrulline) amateur male runners performed two half-marathon races. Jump height, heart rate and rating of perceived exertion were evaluated before and after the races. Moreover, muscle soreness and plasma markers of muscle damage and metabolism were evaluated for 72 h after the races.

**Results**: Muscle soreness perception was significantly lower from 24 to 72 h after the race with CWJ beverage. Immediately after the races, runners under CWJ condition showed plasma lactate and glucose concentrations significantly lower and higher lactate dehydrogenase and l-arginine concentration than runners under PLA. A maintenance of jump heights after the races under CWJ supplementation was found, decreasing significantly with PLA.

**Conclusion**: A single Fashion watermelon juice enriched in l-citrulline dose diminished muscle soreness perception from 24 to 72 h after the race and maintained lower concentrations of plasma lactate after an exhausting exercise.

## Introduction

L-Citrulline is a non-essential amino acid, whose main dietary source is watermelon (*Citrullus vulgaris*). l-Citrulline is a potent endogenous precursor of l-arginine, which is a substrate for NO (nitric oxide) synthase (NOS) [1,2]. NOS leads to NO formation from l-arginine and oxygen and generates l-citrulline as a by-product [3]. In sports physiology, NO has also received much interest, because of the ergogenic effect [1] as a potent vasodilator and modulator of mitochondrial respiration during physical exercise [3], increases muscle contractility, muscle repair, muscle blood flow, glucose uptake, and resistance exercise performance [4,5]. Furthermore, l-citrulline is an essential component participating in the urea cycle in the liver. During intense exercise, there is an increase in the production of ammonia and IMP (inosine monophosphate) in the exercised muscle, being linked to the establishment of muscle fatigue. To avoid the accumulation of these compounds and the decrease of cellular pH, the urea cycle in the liver is responsible for ammonia elimination in the form of urea [6]. This is very important, because a high concentration of ammonia in blood results increases the rate of glycolysis [7] and anaerobic glycolysis results in the accumulation of blood lactate and increased fatigue. By buffering ammonia through the urea cycle, citrulline supplementation is expected to enhance the aerobic utilisation of pyruvate and decrease lactate production via the anaerobic pathway. In animal models, citrulline supplementation decreased lactate production [8] and increased the time to exhaustion [6,8].

Additionally, eccentric exercises produce muscular myofibril ruptures, especially at high intensities, causing muscle damage. This damage produces muscular fatigue that limits performance, decreasing the force, the peak power, or/and the speed. Muscle fatigue depends on the intensity, duration, and type of exercise performed [9]. Because of the muscle damage, several myocellular proteins are released into the blood stream increasing the plasma concentrations of myoglobin, creatine kinase (CK), and lactate dehydrogenase (LDH) [10]. Thus, the serum level of these muscle damage markers shows the functional status of muscle tissue, and varies widely in both pathological and physiological conditions [11].

Scientists and coaches are continually looking for techniques in order to develop more effective and efficient methods to improve exercise performance [12]. One of the popular methods commonly used by athletes to maximise their physical performance is intake of legal ergogenic aids [13]. Previous studies have demonstrated the positive ergogenic effects of l-citrulline supplementation in anaerobic exercise [14,15]. In this way, l-citrulline-malate supplementation (8 g) increased submaximal resistance exercise performance (60–80% 1RM) [16,17], decreased rating of perceived exertion (RPE) [4], and increased the heart rate (HR) response [18]. In addition, watermelon juice (1.2 g of l-citrulline) and enriched watermelon juice (6 g l-citrulline) were reported as decreasing muscle soreness after 24 h of exercise [19]. However, although the response to l-citrulline in anaerobic exercise is known, the mechanisms of this aid in aerobic exercise remain unclear. The results of studies which analysed the response of this substance are controversial. For example, Cutrufello et al. showed no changes in aerobic performance and physiological variables in laboratory conditions after a single dose of l-citrulline (6 g) or watermelon juice (~ 1.0 g l-citrulline) as a pre-exercise supplement in healthy male subjects [20]. In addition, Bailey et al. observed no improvement in time to exhaustion during severe-intensity exercise after 16 days of watermelon juice supplementation (3.4 g of l-citrulline per day) [21]. However, Hickner et al. found an increase in aerobic performance with a reduction in treadmill test time when 9 g of l-citrulline were ingested 24 h before testing in healthy male subjects under laboratory conditions [22]. Moreover, Bailey et al. observed an improvement in blood pressure, VO_2_ kinetics, and cycling exercise performance in healthy adults after seven days of l-citrulline (6 g) [23]. Therefore, the aim of this investigation was to analyse the ergogenic effects (as enhancing physical performance) of an acute dose of 500 mL of Fashion watermelon juice enrichment with 3 g of l-citrulline (CWJ) on physical performance during a half-marathon race in trained runners.

## Material and methods

### Participants

Twenty-one healthy, amateur male runners (age: 35.3 ± 11.4 years; height: 175.5 ± 7.6 cm; weight: 73.6 ± 9.1 kg; muscle mass: 14.1 ± 5.4%; VO_2_ max: 56.3 ± 7.2 mL kg^−1^ min^−1^), with at least four years of experience in endurance events, participated in this study. All participants reported that they aerobically trained for 5.1 ± 1.5 days of training per week, 9.8 ± 1.4 weekly training hours, and 78.8 ± 12.4 min of daily training. Inclusion criteria were the following: 18–45 years old, without any musculoskeletal disorder within six months before the study; also, any subject reporting certain lifestyle factors and diseases that decrease NO production was excluded from participation; subjects consuming any supplements within the last year (branched-chain amino acids, protein, l-arginine, l-citrulline) were excluded from participation. Prior to testing, the experimental procedures, risks, and discomforts associated with the study were explained to all subjects and they provided signed informed consent, approved by the University’s Institutional Review Board and in accordance with the Declaration of Helsinki.

### Study design and beverages tested

Watermelon juice from Fashion watermelon cultivar enriched in l-citrulline (3.45 g per 500 mL) was evaluated with respect to a placebo beverage (without l-citrulline). The placebo and the external addition of l-citrulline (3.0 g per 500 mL) to Fashion watermelon juices were made in the pilot plant of UPCT (Cartagena, Murcia, Spain). The characteristics of different beverages are shown in Table 1. l-Citrulline, pH, titratable acidity (TA), and total soluble solids (TSS) were determined according to Tarazona et al. [19]. A randomised, double blind, crossover design where participants served as their own controls was used. After consuming a standardised breakfast (3 h) and 2 h before the onset of the race, each participant consumed either Fashion watermelon cultivar enriched in l-citrulline (CWJ) or placebo (PLA) in randomised order. After consuming the juice, participants underwent a 1 h seated rest period.

**Table 1.** Physicochemical characteristics and content of bioactive compounds in the different beverages.

	Placebo	Watermelon juice +l-citrulline
TSS (ºBrix)	7.30 ± 0.10	9.00 ± 0.10
TA (malic acid g 100 mL^−1^)	0.21 ± 0.00	0.25 ± 0.00
Lycopene (mg L^−1^)	ND	13.98 ± 0.65
l-Citrulline (g L^−1^)	ND	6.91 ± .021

Total Soluble Solids (TSS); Titratable acidity (TA); ND, not detected. Values are means (n = 3) ± SD.

Each participant performed two half-marathons separated by two weeks to allow their recovery between the trials. Both races were held at the same time of the day. In the first half-marathon, participants performed 21,097 m with 114 m of accumulative altitude, 62 m were uphill and 52 m were downhill. In the second one, participants performed 21,097 m with 131 m of accumulative altitude, 61 m were uphill and 70 m were downhill. The mean temperature in each event was 25.7°C during the first trial and 24.8°C during the second one. Subjects did not ingest caffeine or alcohol at least 24 h before each testing session and performed the last exhaustive training at least 48 h before the evaluation. Subjects were required to ingest a diet rich in carbohydrates at least 3 h before the start of testing. The food items and total amount of water intake for 24 h prior to each trial, during each event, and 72 h before the half-marathon by the subjects were recorded using an individualised food logbook for nutrition recall. Participants were asked to replicate the first trial’s dietary intake for the second trial.

### Squat jump (SJ) and countermovement jump (CMJ) test

Thirty minutes before the onset of the race, participants arrived at the start line, anthropometric measures were taken, and jump height tests were performed. Body composition (muscle mass and body fat percentage) was assessed with a segmental multifrequency bioimpedance analyser, which uses an eight-point tactile electrode method (Tanita BC-601, Tanita Corp, Japan). Measurements were obtained as described by the manufacturer and the system was calibrated prior to each testing session. Body mass and stature were measured using Seca 700 (Seca Ltd., Germany) scale. Subsequently, after their habitual warm-up the jumping ability of the athletes was evaluated with a force platform (Kistler 9286AA Portable, Kistler, Switzerland) with the sampling rate set at 1000 Hz. The subjects performed two different jumps, a squat jump (SJ) and a countermovement jump (CMJ). The arms were kept at the waist all the time to minimise any extra contribution to the jump impulse by the upper body. Each jump type was repeated three times, and the best result was used. A 2 min rest was allowed between jumps to minimise the effect of fatigue on jump performance. The SJs were performed starting from a 90º knee angle position, and no drop or countermovement was permitted. For the CMJs, the subjects were instructed to perform the jump as fast as possible with the aim to activate the stretch-shortening cycle Jump heights (*h*) were calculated from the take-off vertical velocity (*v*) and gravity (*g*) using the following equation: *h* = *v*_i_^2^ · 2*g*^−1^. Jump height was determined in SJ and CMJ and expressed in cm.

### Evaluation of physical performance

Heart rate (HR) was recorded (Polar RS800; Polar Electro Oy; Kempele, Finland) during the entire race. Maximum and average HR of each trial were determined. Rating of perceived exertion (RPE) was measured after each competition using a 6–20 RPE scale [24]. Muscle soreness for lower limbs was evaluated using a 1–5 scale immediately after, 24, 48, and 72 h after the races [16,19].

Finally, within 3 min after the race, participants went to a finish area and performed vertical jumps as previously described. Then, participants rested for 5 min and a venous blood sample was obtained using the procedures described above.

### Plasma analyses

Blood samples (11 mL) were taken from each subject 1 day before exercise (basal), immediately after exercise, and 24, 48, and 72 h post exercise. Venous blood samples were collected from each subject by antecubital venepuncture with vacutainer system to determine the basic biochemistry, contained in arginine and muscle damage markers. After making withdrawals, samples were kept at 2°C. It was expected to take at least 30 min until complete blood coagulation. Samples were centrifuged for 10 min at 3800 rpm to separate formed elements and fibrin clot, and supernatants were recovered for further analyses following the sanitary procedures.

l-Arginine was determined as described by Collins et al. [25]. An aliquot (40 µL) of plasma was mixed with 40 µL of 1.5 M HClO_4_ to precipitate proteins. To this solution, 900 µL of HPLC-grade water and 20 µL of 2 M K_2_CO3 were added. The mixture was centrifuged at 10,000*g* for 1 min and 100 µL of the supernatant was injected into a liquid chromatograph (HPLC, Waters, Milford, MA, USA) with fluorescent detector (Agile serie 1200). Arginine was quantified by comparison with an external standard of arginine (Sigma Chemicals, Madrid, Missouri, USA) and results were expressed in mg per dL. Lactate and C reactive protein (CRP) were determined by molecular absorption spectrometer and immunoturbidimetry, respectively, by clinical chemistry system (ADVIA ® 1800, Siemens, Japan). The rest of the serum biochemical analytes were measured using an autoanalyser Spinteach 640 (Spinreact, Girona, Spain); reagents and chemicals were supplied with the purchased commercial kits (Spinreact, Girona, Spain). The different methods used for analysis of biochemical analytes were as follows: (1) determination of blood enzymes conducted using aspartate transaminase (AST) by the International Federation of Clinical Chemistry (IFCC) enzymatic-UV method, alanine transaminase (ALT) by the IFCC enzymatic-UV method, LDH by the German Society of Clinical Chemistry (*Deutsch Gesellschaft für Klinische Chemie*, DGKC) kinetic-UV method, and CK by the *N*-acetylcysteine (NAC) kinetic-UV method and the results are expressed in U per L; (2) glucose by glucose oxidase-peroxidase enzymatic colorimetric method; (3) cholesterol by cholesterol oxidase and peroxidase enzymatic colorimetric method; (4) creatinine by Jaffé colorimetric kinetic method; (5) urea by urease-glutamate dehydrogenase kinetic method; (6) uric acid by uricase-peroxidase enzymatic colorimetric method; (7) myoglobin by turbilatex myoglobin latex turbidimetry; (8) ferritin by turbilatex ferritin latex turbidimetry. Glucose, creatinine, urea, uric acid, cholesterol, lactate, and CRP were quantified in mg per dL, while myoglobin and ferritin were quantified in ng per mL.

### Statistical analysis

Statistical analysis was performed using the statistical program SPSS (SPSS 22 for Windows, SPSS Inc. Chicago IL.). The distribution of data was initially verified by the Shapiro–Wilk test. Depending on data normality, analysis of variance (ANOVA) with pairwise comparisons post hoc test using the Bonferroni corrections (SJ, CMJ, hear rate, race time) was used or Friedman with Wilcoxon post hoc test was performed with the Bonferroni corrections (RPE, muscle soreness, parameters blood test: arginine, myoglobin, ferritin, C-reactive protein, AST-GOT, ALT-GPT, LDH, CPK, lactate, creatinine, uric acid, urea, glucose, and cholesterol) in not normal data; *p* ≤ 0.05 was considered statistically significant. Data are presented as mean ± standard deviation (SD).

## Results

### Effect of CWJ on time trial, HR, and jump heights

For the half-marathon time trial, no significant reduction was found in the volunteers that drank PLA (99.9 ± 13.5 min) compared with CWJ (99.9 ± 11.9 min). Moreover, no significant differences in the average HR were observed between PLA (162.8 ± 9.7 beats min^−1^) and CWJ (165.7 ± 7.5 beats min^−1^). Similar results were found in the maximum HR (179.1 ± 10.6 beats min^−1^ and 182.4 ± 13.1 beats min^−1^, PLA and CWJ, respectively).

**Figure 1.**
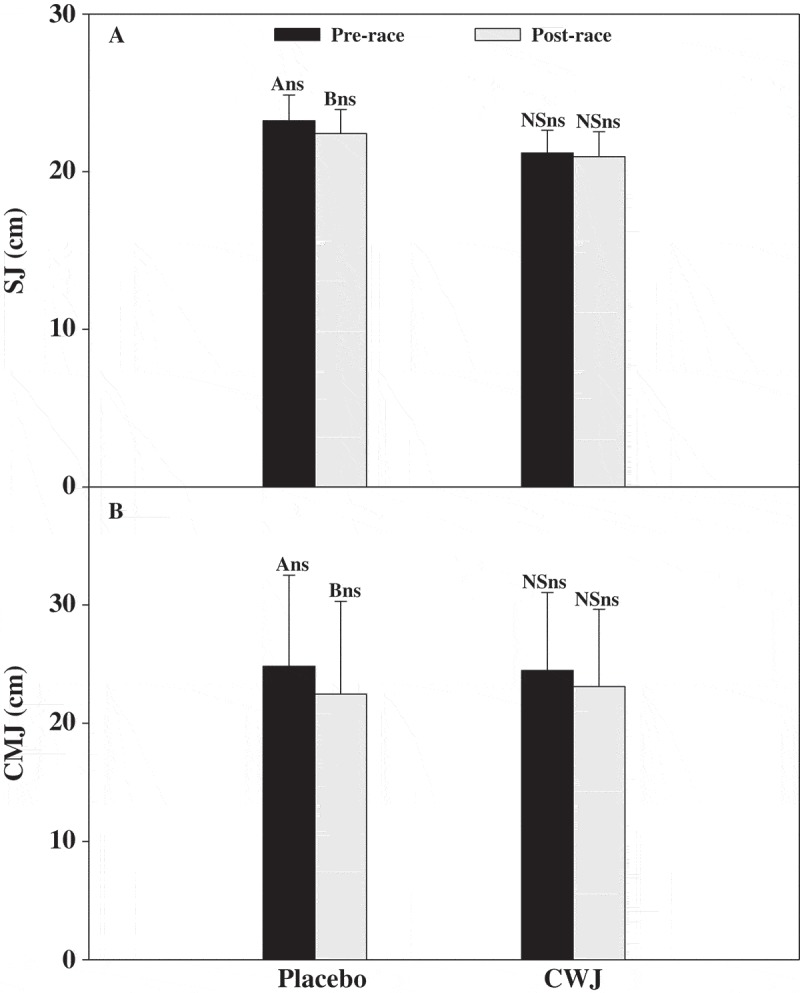
Effect of Fashion watermelon juice enriched in l-citrulline (CWJ) on jump recovery on squat jump (SJ) and countermovement jump (CMJ). Different capital letters for the same beverage show significant differences between the times and different lower case letters for the same time show significant differences between beverages.

On the other hand, SJ and CMJ heights decreased significantly, around 9% (ΔhSJ −2.3 cm) and 10% (ΔhCMJ −2.4 cm), respectively, after the race under the PLA beverage. In contrast, when participants were supplied with CWJ beverage no significant differences were observed between before and after the race in both jump heights (ΔhSJ: −1.5 cm; ΔhCMJ: −1.4 cm) (Figure 1).

### Effect of CWJ on RPE and muscle soreness perception

The RPE values, immediately after each half-marathon, showed no significant differences between the beverages tested (15.4 ± 1.9 and 15.4 ± 1.8 in PLA and CWJ, respectively).

**Figure 2.**
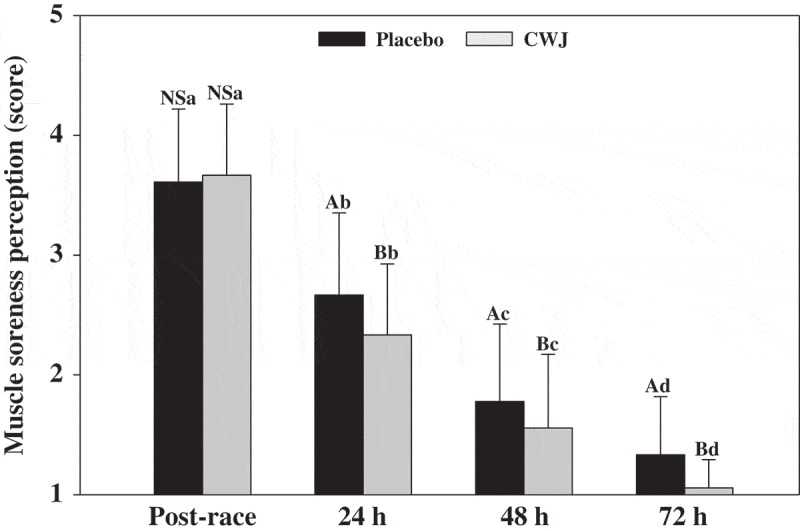
Effect of Fashion watermelon juice enriched in l-citrulline (CWJ) on muscle soreness at 30 min, 24 h, 48 h, and 72 h after half-marathon. Different capital letters for the same time show significant differences between beverages and different lower case letters for the same beverage show significant differences between the times.

On the other hand, muscle soreness perception immediately after half-marathons were similar in both beverages tested (Figure 2). However, at 24 h post exercise a significant decrease in muscle soreness perception was observed in runners under CWJ compared to PLA. Moreover, a significant decrease during 72 h after half-marathon was observed in both beverages, maintaining the significant differences between beverages during this time (Figure 2). Additionally, at 24 h after half-marathon, around 5% of participants who took CWJ scored an absence of soreness, and non-acute pain with negative interference in the immediate moderate training was reported by participants. On the contrary, at 24 h after half-marathon participant that took PLA no scored an absence of soreness and 11% participants reported an acute pain with negative interference in the immediate moderate training (data not shown). At 48 h after half-marathon, CWJ presented an absence of soreness in 50% of participants compared to 33% in PLA. Finally, at 72 h from half-marathon, around 95% of participants indicated an absence of soreness when they took the CWJ compared to 67% for PLA (data not shown).

### Effect of CWJ on blood biomarkers

**Figure 3.**
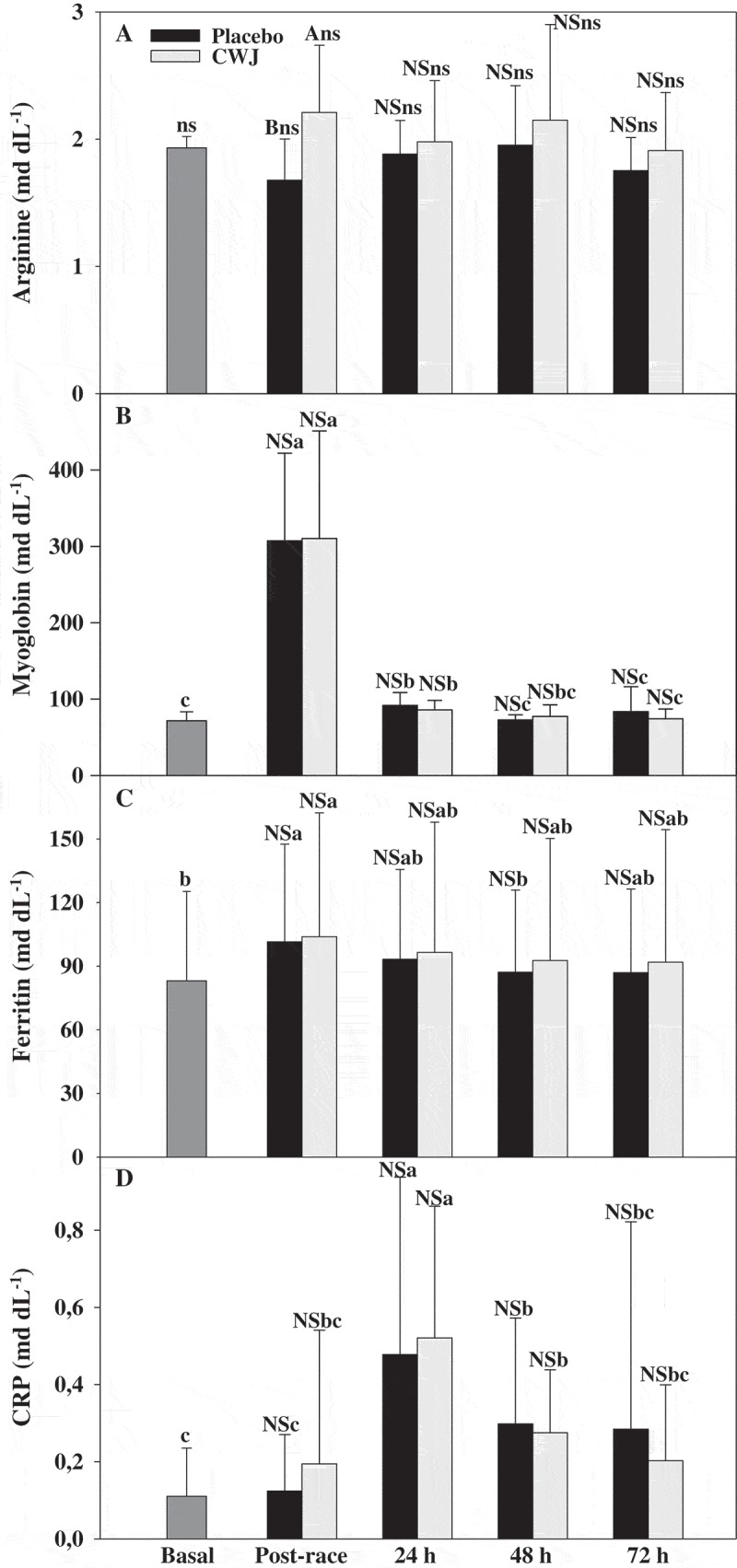
Effect of Fashion watermelon juice enriched in l-citrulline (CWJ) on blood parameters such as arginine, myoglobin, ferritin, and C-reactive protein (CRP) after half-marathon. Different capital letters for the same time show significant differences between beverages and different lower case letters for the same beverage show significant differences between the times.

After the race, participants who took CWJ beverage increased the plasma concentrations of arginine around 27% respect to PLA and 14% respect to basal levels, while PLA showed a decrease of around 13% respect to basal levels (Figure 3(a)). However, only significant differences were detected between CWJ and PLA after the race (Figure 3(a)).

On the other hand, after races myoglobin and ferritin increased respect to basal levels, although no significant differences were observed between beverages (Figure 3(b,c)). Moreover, myoglobin levels were above the basal levels during 24 h after races with the highest values after races (328% and 332% in PLA and CWJ, respectively) while ferritin levels were only at post-race (22% and 25% in PLA and CWJ, respectively), and both parameter levels were reconstituted at 48 and 24 h after races (Figure 3(b,c)). In addition, CRP showed a trend to increase after half-marathons. Significant differences were observed 24 h after races (332% and 371% in PLA and CWJ, respectively) respect to basal levels, without significant differences between beverages and CRP values were normalised at 72 h after races (Figure 3(d)).

**Figure 4.**
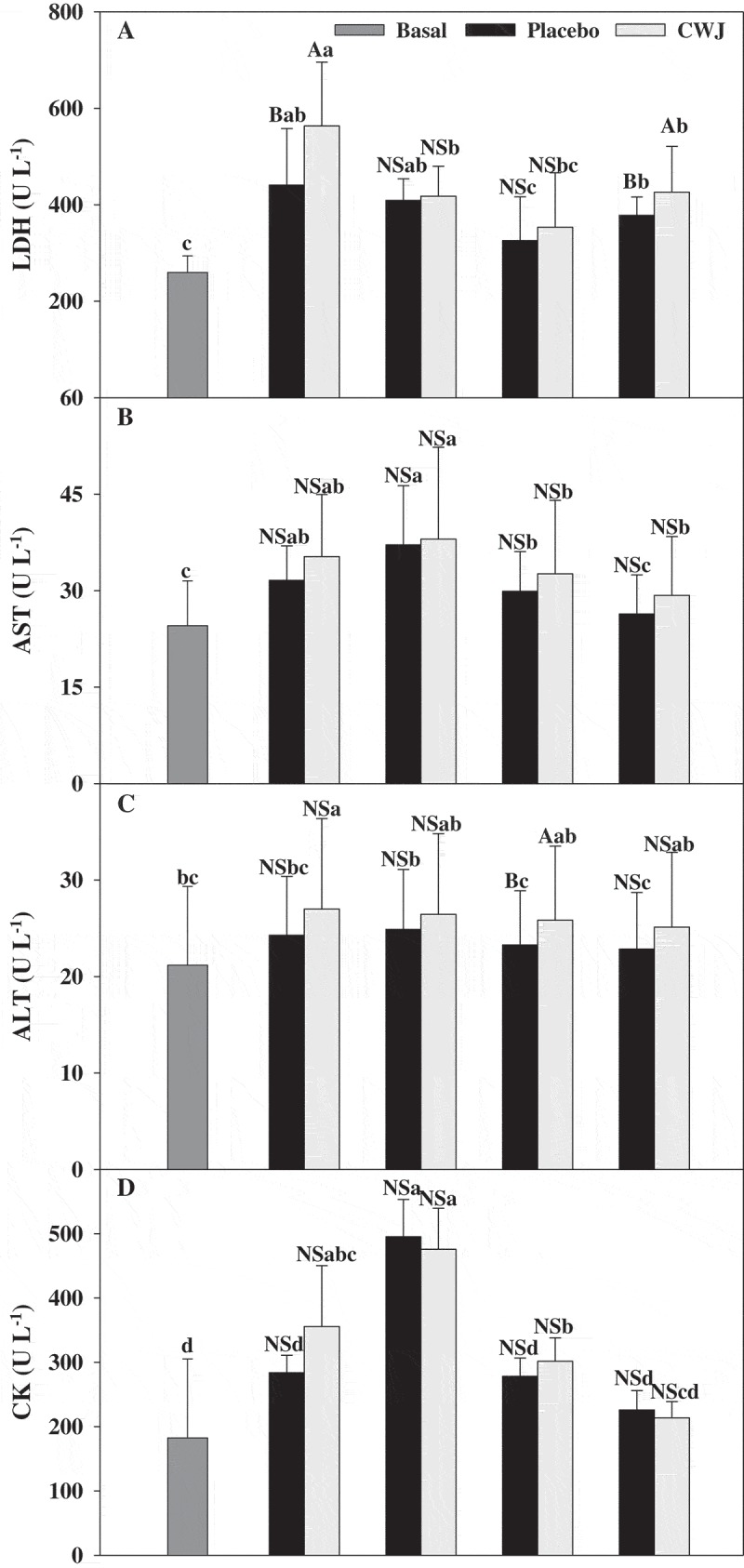
Effect of Fashion watermelon juice enriched in l-citrulline (CWJ) on blood enzymes such as glutamic-oxaloacetic transaminase (GOT), glutamic-pyruvic transaminase (GPT), lactate dehydrogenase (LDH), and creatine phosphokinase (CPK) after half-marathon. Different capital letters for the same time show significant differences between beverages and different lower case letters for the same beverage show significant differences between the times.

After half-marathons, the plasma levels of these enzymes (LDH, AST, ALT, and CK) showed an increase (Figure 4). Although volunteers who drank CWJ showed higher values than PLA, no significant differences were observed between beverages except in LDH. After races, this enzyme suffered a significant increase, around 117% in athletes under CWJ and 70% in PLA respect to basal levels (Figure 4(a)). AST plasma levels showed a tendency to increase during the 24 h after the race (51% and 55% in PLA and CWJ, respectively), while ALT showed the maximum levels immediately after the race (15% and 28% in PLA and CWJ, respectively) (Figure 4(b,c)). Then, 48 and 24 h after the races AST and ALT levels, respectively, were normalised. However, plasma concentrations of CK increased after exercise, reaching the maximum levels (increase around 171% and 161% in PLA and CWJ, respectively) at 24 h after the half-marathon and returning to normal levels at 48 h in PLA and 72 h in CWJ (Figure 4(d)).

**Figure 5.**
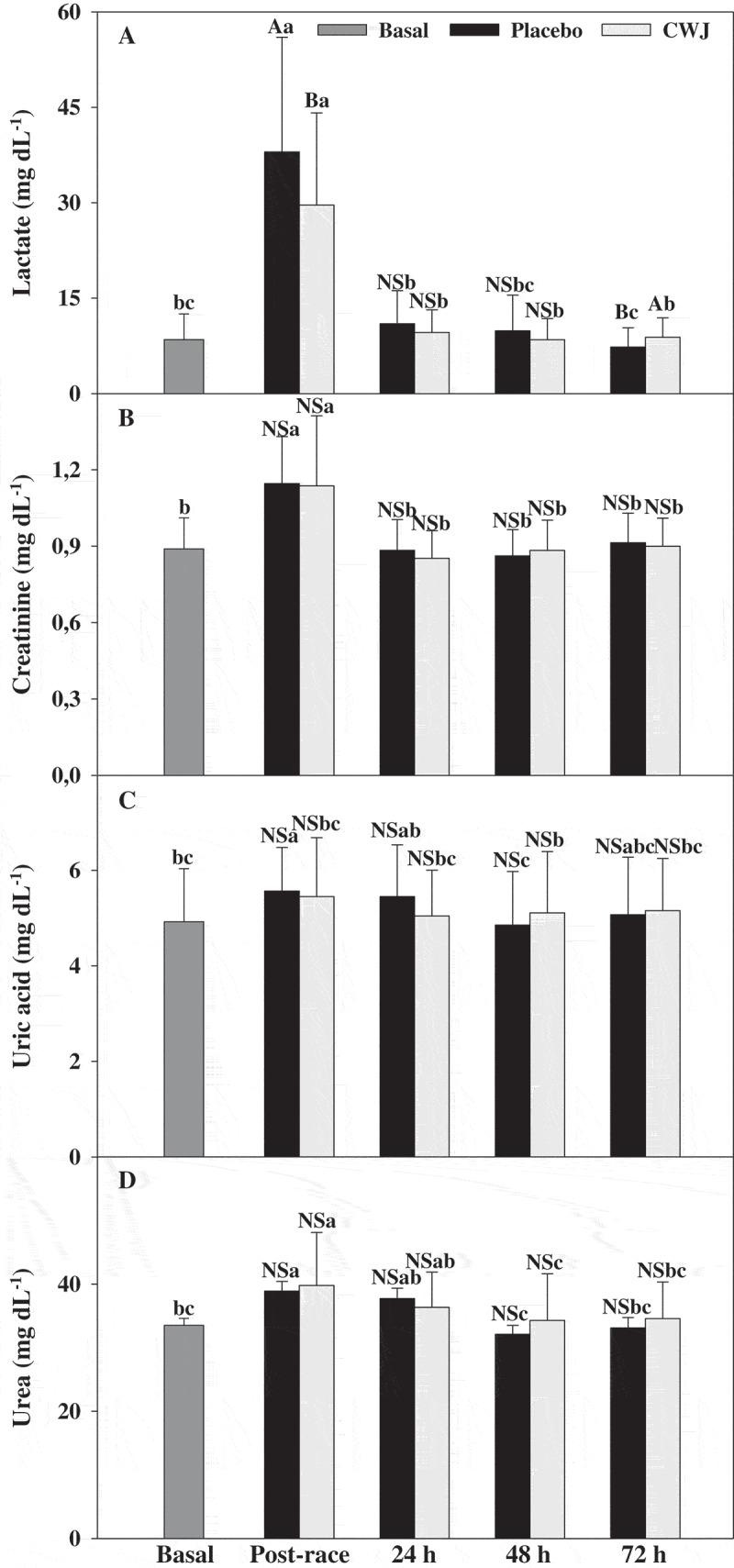
Effect of Fashion watermelon juice enriched in l-citrulline (CWJ) on blood parameters such as lactate, creatinine, uric acid, and urea after half-marathon. Different capital letters for the same time show significant differences between beverages and different lower case letters for the same beverage show significant differences between the times.

On the other hand, plasma lactate levels increased after the half-marathon, being significantly higher in PLA (4.5 times basal levels) than in CWJ (3.5 times basal levels) (Figure 5(a)). Lactate concentration increases after exercise, but as expected at 24 h, the concentration decreased to baseline levels in all volunteers. Moreover, post-race serum creatinine, plasma uric acid and urea levels were higher than basal levels, although no significant differences were observed between beverages and levels were restored 24 h after the half-marathon (Figure 5(b-d)).

**Figure 6.**
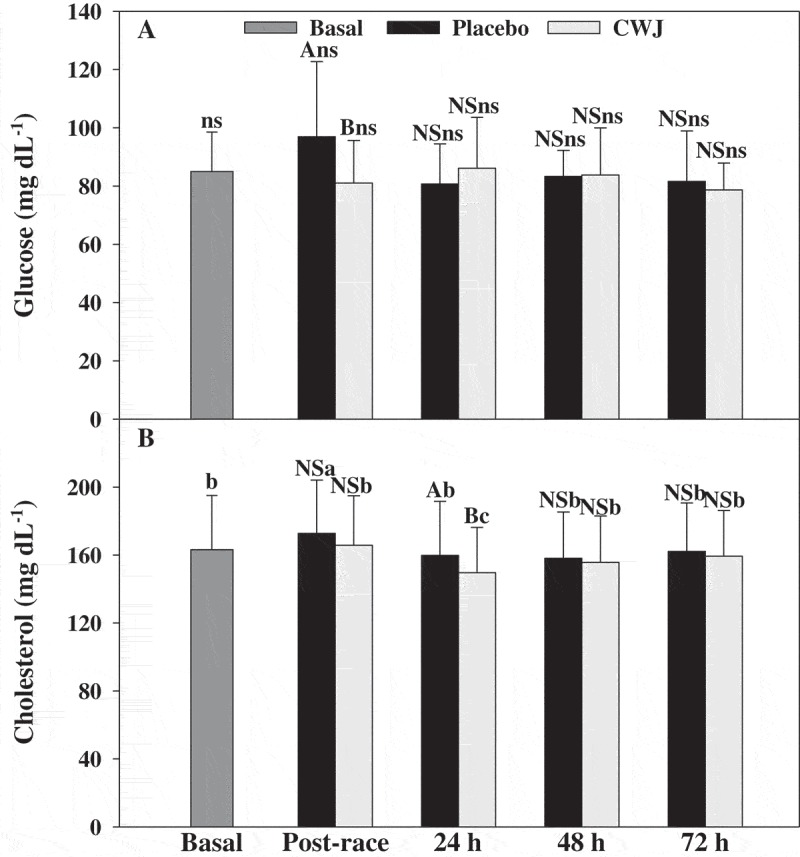
Effect of Fashion watermelon juice enriched in l-citrulline (CWJ) on blood substrates such fasting glucose and cholesterol after half-marathon. Different capital letters for the same time show significant differences between beverages and different lower case letters for the same beverage show significant differences between the times.

Finally, post-race plasma glucose concentrations were significantly higher in PLA (increased 14% respect to basal levels) than CWJ (decreased 5% respect to basal levels), although similar with respect to baseline levels (Figure 6(a)). The concentrations of plasma cholesterol were around 6% higher in PLA immediately after races than basal levels, although CWJ post-race concentrations of plasma cholesterol did not show significant differences respect to basal levels and to PLA post- race levels (Figure 6(b)). However, at 24 h after races PLA showed significantly higher cholesterol levels than CWJ with a significant decrease in CWJ respect to basal levels (Figure 6(b)). The glucose and cholesterol concentrations were re-established at 24 and 48 h, respectively.

## Discussion

In our experiment, participants showed a similar time for each half-marathon not showing significant differences between PLA and CWJ. Cutrufello et al. reported similar results during an aerobic incremental test to exhaustion after l-citrulline supplementation (1–6 g) [20]. Although the aerobic performance was analysed in the study of Cutrufello et al [20], must be taken into account that the subjects performed a Bruce test with ~ 10 min of time trial. Bailey et al. also reported that volunteers did not improve time-to-exhaustion during severe-intensity exercise (a ramp incremental cycle test on an electronically-braked cycle ergometer) after receiving 16 days of supplementation with 300 mL day^−1^ of a watermelon juice concentrate (∼3.4 g l-citrulline day^−1^) [21]. On the other hand, Hickner et al. found an improvement in time to exhaustion, during an aerobic incremental test, after a dose of 3 or 9 g of l-citrulline in active subjects [22]. In the test protocol used in this study [21], each subject ran for approximately 15 min on a motorised treadmill while the speed and the grade increased until athletes could no longer continue. In addition, citrulline or citrulline malate as supplements to increase NO production do not act as stimulants and not affect HR during exercise [4]. For this reason, the HR was similar in both beverages tested, PLA and CWJ. Similar results were found in volunteers submitted to a cycle ergometer test who took enriched watermelon juice with l-citrulline respect to PLA [19].

The RPE is one of the most important limiting factors of performance in endurance events with similar duration. In this study, the RPE values in both trials showed that the intensity is close to the individual anaerobic threshold of the subjects [26,27]. There has been no detailed investigation of changes in RPE after l-citrulline supplementation in long duration exercises. However, similar RPE values were found in a 12 min intervallic test in trained participants [19].

Armstrong [28] identified a decrease in muscle contractility due to muscle damage in endurance races with metabolic overload and high mechanical stress. Explosive muscle actions, like those used in the jump, can also be impaired by muscle damage [29]. In this way, endurance races could decrease the vertical jump performance and jump may be used as an indicator of fatigue [30]. Several studies have shown the relationship between muscle damage and jump height after long endurance races [31,32]. Race performance has been correlated with leg muscle power during CMJ and with the running pace of the race [33,34]. Additionally, this study [34] shows that athletes who obtained less decrease in jump height performed the race quickly and with reduced values of muscle damage. Interestingly, in our study, participants supplemented with CWJ showed no significant decrease in jump heights while under PLA beverage the reduction in the performance was significant. Bailey et al. also found over the first 10 s that the all-out effort was higher in the watermelon juice condition [21]. These findings suggest that the athletes supplemented with CWJ may perform the race with a higher intensity and with less fatigue improving their time race. However, in our study no changes were observed in time trial.

l-Citrulline is a precursor of l-arginine, as it can be observed by the increase in plasma l-arginine concentrations after CWJ ingested respect to PLA, which is in agreement with previous studies showing similar results [25,35,36]. Moreover, a significant arginine decrease was observed after half-marathons in PLA respect to CWJ. Sureda et al. observed similar results, when supplementation with citrulline avoided the limited availability of arginine by NOS to increase the NO synthesis [15]. Bailey et al. also confirmed that, compared to control and placebo, plasma l-citrulline, l-arginine, and NO were higher after watermelon juice supplementation [21]. Therefore, the intake of L-citrulline could reduce muscle fatigue and maintain muscle contractility for a longer period of time and improve the exercise performance due to vasodilatory response and blood flow effect of NO [15,37,38]. The significant differences at 24, 48, and 72 h post-races in muscle soreness perception are in accordance with the results of previous studies performed in anaerobic exercises [16,19].

As the rate of glycolysis is increased during high-intensity exercise, anaerobic glycolysis results in the accumulation of blood lactate and increased fatigue. By buffering ammonia through the urea cycle [39], citrulline supplementation is expected to enhance the aerobic utilisation of pyruvate, thus decreasing lactate production via the anaerobic pathway. In our study, plasma lactate levels were lower in CWJ than PLA and Takeda et al. [8] described similar results. The response of lactate concentration in placebo group is associated to exacerbated perturbations of cellular homeostasis in active muscles. Higher blood lactate concentration produces higher blood pH and this fact suggest increased reliance on glycolysis to maintain ATP supply, indicating a greater anaerobic energy release without CWJ. Therefore, when aerobic metabolism is not capable of meeting ATP demand, the activation of anaerobic glycolysis can be further elevated to meet the short-term requirements for ATP [40]. Moreover, Bendahan et al. demonstrated that citrulline malate ingestion reduces the sensation of fatigue, increase the rate of oxidative ATP production during exercise and the rate of phosphocreatine recovery after exercise, indicating a larger contribution of oxidative ATP synthesis to energy production [14]. Furthermore, l-citrulline can promote aerobic energy production, reduces fatigue perception and increases the recovery rate [14]. LDH interconverts pyruvate and lactate with concomitant interconversion of nicotinamide adenine dinucleotide (NAD) and its reduced form NADH. However, there are five isoenzymes (LDH1, LDH2, LDH3, LDH4, LDH5), made of the combination between M-polypeptide (catalyse the conversion of pyruvate to lactate) and H-polypeptide (improve the aerobic oxidation of pyruvate); therefore, the anaerobic or aerobic pathway depends on the number of each chain present in the active LDH isoenzyme [11]. LDH catalyses the conversion of pyruvate to lactate and improves the aerobic oxidation of pyruvate [11]. In this way, the higher plasma LDH concentration observed in CWJ respect to PLA after half-marathon races may be due to the higher activity of this enzyme enhanced by the l-citrulline intake increasing aerobic pathway.

During exercise, muscle cells obtain energy via several pathways, which involve ATP-phosphocreatine (PCr) system, anaerobic glycolysis, tricarboxylic acids (TCA) cycle, and electron transfer system [41]. Arginine is a precursor in the renal synthesis of creatine, which is an important constituent of skeletal muscle. Sureda et al. described that citrulline supplementation could increase the whole body nitrogen availability to allow higher protein synthesis and to increase the protein content in muscle during exercise, enhancing the use of amino acids [15]. It could explain the higher CK level respect to basal levels in CWJ although without significant differences with PLA. In our study, Leers et al. previously described the significant increase in creatinine after the race in an experiment with 27 amateur runners, accompanied by increased urea immediately after the race which normalised 1 day later [42]. During physical exercises of high intensity and short duration, PCr is the energy substrate, and the product of their degradation is creatinine [43].

In the experiment, uric acid increased after the race in PLA respect to basal levels, probably because of increased ATP catabolism resulting from the exhaustive exercise [44]. Exercise may require an increase in the activity of adenylate cyclase or myokinase acting as an additional source of energy by producing 1ATP and 1AMP from 2 ADP [45]. While ATP is used for energy, the AMP is degraded to IMP which is degraded to hypoxanthine, xanthine and finally to uric acid [45]. Xanthine oxidase (XOD) utilises hypoxanthine or xanthine as a substrate and O_2_ as a cofactor to produce superoxide (·O_2_^−^) and uric acid, while xanthine dehydrogenase (XDH) acts on these same substrates but utilises NAD as a cofactor to produce NADH instead of O_2_^−^ and uric acid, and in an intensive exercise XOD would be the enzyme responsible for contributing to oxidative stress during exercise [46].

Regarding parameters of AST, ALT, myoglobin, ferritin, and CRP, no significant differences were found between the beverages tested and this could be associated to the high intensity of half-marathon exercise, which diminished the potential differences between beverages. Levels of enzymes such as CK, LDH, AST, and ALT (all present in muscle fibres) are elevated in conditions when muscle damage is present [47]. In our experiment, LDH, AST, and CK enzymes increased after the race (post-race and/or after 24 or 48 h), which supports the notion that the high-intensity exercise used in this study resulted in skeletal muscle injury as Machado et al. [48] found in soccer players. Munjal et al. observed post run levels of CK, LDH, and myoglobin significantly higher than the pre-run levels as the result of a moderate (5–10 mile run) exercise [49]. In fact, and agreeing with Lippi et al., myoglobin showed a more rapid release from the injured tissue, reaching an earlier (and higher) peak in plasma and returns to normal values much faster than CK, LDH, and AST [50]. Accordingly, the larger increase in myoglobin can be explained by the overall faster elimination kinetics [51].

On the other hand, our CWJ supplementation reduced plasma glucose and cholesterol concentrations compared to PLA. Not significant differences in the plasma glucose levels after the treadmill exercise were observed by Hickner et al., between placebo and different doses of l-citrulline (3 g or 9 g), describing a glucose intake dependent on contraction and a reduction in insulin response to this high-intensity exercise [21].

## Conclusions

A single Fashion watermelon juice enriched in l-citrulline dose increased plasma l-arginine concentrations, diminished muscle soreness perception from 24 to 72 h after the race, and enhanced aerobic pathways maintaining lower concentrations of plasma lactate and increasing the activity of LDH. In addition, this ergogenic aid produced a maintenance of jump heights after the races under CWJ supplementation. The results of this study may be useful for coaches and athletes in order to improve the aerobic pathway during competition and the recovery perception after long duration races. In addition, future research about the effects of l-citrulline for longer supplementation periods should be considered to evaluate its impact on improving the ergogenic aid of Fashion watermelon juice.
